# The Causes of Hernia—With a New Theory

**Published:** 1894-10

**Authors:** Byron Robinson

**Affiliations:** 34 Washington Street; Chicago


Buffalo Medical and Surgical Journal

VOL. XXXIV. OCTOBER, 1894. NO. 3.

### Original Communications.

### THE CAUSES OF HERNIA—WITH A NEW THEORY.

By BYRON ROBINSON, of Chicago.

TO ONE who does nothing but abdominal surgery and gynecology it is of interest to note what produces hernia. Since man began to stand erect there can be but little doubt that he has suffered from rupture of some portion of the abdominal wall. Hernia has been attributed to innumerable causes, but the physician who thinks for himself perceives that hernia can be due simply to no one cause. Anyone who has observed hernia for any length of time must plainly see that it is a slow, insidious process. It may manifest itself suddenly, but that is only the end of a long persistent force. For the past five years I have paid some attention to the causes of hernia, its anatomical nature and radical operation for cure. On the living, hernia can be felt and seen ; on the dead, by slow, careful dissection one can observe the passage through which the sac and its contents pass out of the abdominal cavity. By the radical operation one can observe how hernia may be cured. One must pass through these stages to well comprehend hernia. Death and failure in surgery always stimulates to better work, and anyone who does much abdominal work is sure to have both death and failure. I will enumerate some well-recognized causes of hernia and will also add one which I have not noticed in the books nor heard discussed. I gained the knowledge from the abdominal drain-tube.

1. The first and most general proposition is that hernia is due to deficiency of the anatomical structures around the hernial orifices. As one can notice, some people have small or deficient viscera, while others may have defects in the abdominal walls. The walls may be locally thin and deficiently closed at the apertures.

2. Most hernia is of congenital origin. The tunica vaginalis may remain open and allow easier descent of the viscera.

3. Heredity is said to play a rôle, but I can only see a cause in heredity on account of producing constitutional defects, weak and deficient anatomical structures. The hernia itself cannot be transmitted.

4. Intra-abdominal pressure is one of the great causes of hernia. It is well recognized that hard or heavy laborers are frequently afflicted with hernia. This is owing to the cause that hard or heavy lifting increases intra-abdominal pressure. Fatty deposits diminish the abdominal cavity and hernia is liable to increase from intra-abdominal pressure.

5. A prize essay was gained by a man who wrote that hernia was caused by swaddling clothes and high breeches. However, this was a long time ago.

6. Another man, a French servant, said it was due to a vegetable diet which tended to elongate the cecum. Never forget that this view is now exactly forty-eight years old.

7. Diseases of the upper air-passages are said to cause hernia. This would be due to cough, which simply increases intra-abdominal pressure.

8. Post-operative hernia is due to deficient healing of the essential anatomical structures, *e. g.*, in the post-operative hernia in laparatomy the tendon produced by the combined blending of the tendons of the external, internal oblique and transversalis muscles, does not unite properly. Age and malignancy I found tended toward deficient union in laparatomy.

9. Irregular action of the gut-wall is the one cause which I wish to add. In laparatomy I use an aluminum drain-tube perforated by quite small holes. The holes are only about one-half as large as those in Keith's glass drain-tubes. The holes are so small that one cannot push a piece of bowel into one. Now, in a series of 100 laparatomies, Dr. Waite and myself have had three cases where the gut-wall has penetrated these small holes in the side of the aluminum drain-tube. We were obliged to steadily pull the tube upward and then tie the bowel wall with a ligature. In one case of Dr. Waite's the drain-tube happened to be a glass tube of Dr. Keith's, and we were obliged to break the tube to safely liberate the gut-wall. Now, it appears to me that the hernia of the gut-wall in these tubal holes is due to irregular action of the muscles in the gut-wall. It is disordered peristalsis. The presence of the tube may irritate the muscles of the gut to act irregular or in a disordered manner. It seems to me the same views can be applied in many cases of hernia. As the gut-wall approaches the small inguinal, femoral or the obturator canal, it may become stimulated to irregular action just as it does around the hole in the drain-tube. In my opinion this explanation will account for the fact that the gut is found protruding several inches through a very small aperture. Besides, the strangulated point will be found one to three inches from the end of the projecting loop. I have wondered almost as much how a loop of bowel could insinuate itself into such a small hole in the abdominal wall, as it could insinuate itself into a small round hole in my aluminum drain-tube. Of course, Keith's glass drain-tube has unnecessarily large holes, and in many cases it is common to find omentum, appendix epiploicus or bowel-wall engaged and even strangulated through one or more of these holes. In my aluminum drain-tube I especially made the side perforations small to avoid the liability to hernia. Hernia through a small hole in the abdominal wall is likely produced from irregular action of the gut-wall, just as invagination is due to irregular muscular action of the bowel-wall.

10. For a long time, and by experiment on quite a number of bodies, I thought that hernia was due to elongated mesentery or unduly long visceral support. But further trial at autopsies on, so far as I could judge, normal viscera has convinced me that I was wrong; for in normal adult bodies the loops of the intestines would reach beyond the hernial orifices without undue stretching.

11. Prolapse of viscera may aid in producing hernia, but so many have prolapsed viscera without hernia that it must be only an indirect cause. I have noted prolapse of the viscera especially in old female cadavers.

12. The erect posture is no doubt one of the causes of hernia, as it is rare in animals on four legs.

13. Rapidly repeated gestation induces hernia.

14. Imperfect or late descent of the testicle and potency of Nuck's canal.

The above and many more causes could be adduced as producing hernia, but the general view of hernia is soon obtained from the study of living and dead subjects that the cause of hernia is due to many immediate and remote factors, *e. g.*, in dissecting infant cadavers, male and female, one can find the cord or round ligament taking a straight course. The internal ring is directly behind the external ring. The inguinal canal has no length or obliquity. One ring lies exactly behind the other. In hernia one can find that the internal ring gradually approaches the other until in a hernia of any size the internal and external ring are applied over each other. An exact idea of the cause of hernia will enable one alone to properly operate. The fact must be kept in mind that Nature must be restored. The obliquity of the canal must be restored.

Marcy's operation is based on the fundamental principle that must cure all hernia. Halstead's and Bassini's operation add the idea of the most effectual method of treating the cord. Fabricius's operation enables one to push the femoral artery and vein outward and build a solid buttress by new tissue by suturing Poupart's ligament to the pectoneal fascia. Cause and effect must be studied together. The theory of irregular muscular action of the gut-wall as a cause of hernia I have never seen in literature or heard it discussed; but it seems to me to offer an explanation of some very small tight herniæ. The combination of Marcy's, Bassini's and Halstead's operation for hernia enables one to restore pathologic conditions to natural ones and the use of these new operations on hernia convince me that I can cure cases.

There is some cause at work producing hernia on the right side more than the left. There is almost double the amount of hernia on the right so far as inguinal is concerned. Also the right side preponderates in regard to both femoral and obturator. Late descent of the testicle, excess of exercise and the direction of the mesentery have all been charged with the cause, but acceptable explanations are yet forthcoming.

34 Washington Street.

